# Optimizing Solid-State Mixed-Strain Fermentation of Forage Mulberry for Improved Nutritional Quality via Anti-Nutritional Factor Degradation

**DOI:** 10.3390/ani16152366

**Published:** 2026-08-03

**Authors:** Yanzhen Duan, Na Wen, Daodian Wang, Xingying Yang, Sha Li, Yemei Yang, Wei Yang, Junrong Huang, Wen Yang, Pingping Li

**Affiliations:** Sericultural and Apicultural Research Institute, Yunnan Academy of Agricultural Sciences, Mengzi 661101, China; 18287280385@163.com (Y.D.); wennaa9915@163.com (N.W.); daodianwang2026@163.com (D.W.); xyyang@yaas.org.cn (X.Y.); 15697068796@163.com (S.L.); yemeiyang111@163.com (Y.Y.); yangwei@yaas.org.cn (W.Y.); huangjunrong1981@163.com (J.H.)

**Keywords:** mixed-culture solid-state fermentation, whole-plant forage mulberry, pH-enzyme synergy, nutritional quality, non-grain feed, in vitro digestibility

## Abstract

To improve the feeding value of forage mulberry as a non-grain feed, solid-state mixed fermentation with *Lactobacillus plantarum* and *Pichia membranifaciens* (1:1) was optimized via single-factor tests and an L_9_ (3^4^) orthogonal design. The optimal conditions (7 days, 28 °C, 30% inoculum, 1:1.2 solid-to-water ratio) reduced crude fiber (CF) content by 44.60%, and increased crude protein (CP), crude fat (EE), and total amino acids (TAA) by 35.77%, 68.85%, and 40.85%, respectively (*p* < 0.01). Mechanistically, the organic acids produced by *L. plantarum* lowered the fermentation pH to 4.0–4.5. This pH change was likely associated with the observed 50.84% increase in yeast cellulase activity. Yeast metabolites appeared to promote the proliferation of lactic acid bacteria (LAB). This implies a potential synergistic interaction between the two strains under acidic conditions. The in vitro ruminal dry matter digestibility (DMD) and CP digestibility reached 72.30% and 78.60%, respectively. These values were significantly higher than those of the unfermented control (*p* < 0.01). Therefore, this pH-enzyme synergistic fermentation strategy can effectively degrade anti-nutritional components. It also improves the feeding nutritional quality of mulberry forage for animal production.

## Simple Summary

To develop grain-saving animal feed, this study uses two microbial strains to ferment mulberry roughage and finds the best fermentation parameters through multi-group experiments. After seven days of fermentation under proper temperature, inoculation amount and moisture proportion, the coarse hard fiber in mulberry is greatly reduced, while protein, fat and total amino acid nutrients rise sharply. One strain produces acidic substances to create a weakly acidic environment. This environment enhances the fiber-degrading capacity of the other strain, and their metabolites support each other’s growth. Fermented mulberry also achieves much higher digestion efficiency in simulated animal stomach environments than untreated raw mulberry. This cooperative fermentation method efficiently removes substances hindering animal digestion and greatly upgrades the nutritional quality of mulberry feed. The research helps cut reliance on grain feed for livestock breeding, saves food resources and supports stable, low-cost animal husbandry development.

## 1. Introduction

With the expansion of animal husbandry (AH) in China, national feed demand has experienced sustained growth. The output of natural forage and dedicated feed crops cannot satisfy livestock nutritional requirements, resulting in heavy reliance on imported feed supplies for animal production across many nations. In 2024, China imported 105 million tons of soybeans, representing a 6.5% year-over-year increase and accounting for 66% of the country’s total grain imports. Against this backdrop, exploring high-protein feed substitutes to replace soybean meal has become an urgent priority to mitigate grain competition between human consumption and livestock production, as well as to curb rising feed costs [1,2].

Mulberry has gained substantial research interest in recent years as an alternative feed resource for ruminants to alleviate the shortage of conventional protein raw materials, owing to its abundant bioactive compounds, CP, amino acids, vitamins and mineral elements [3]. Furthermore, mulberry exhibits extensive ecological adaptability and strong tolerance to adverse environmental conditions, including drought, high temperature, salinity and heavy metal stress, which endows it great potential for large-scale exploitation [4]. Nevertheless, mulberry forage inherently contains anti-nutritional factors (ANFs) such as tannins and lignin. Most monogastric animals lack endogenous cellulase and ligninase; direct feeding of untreated mulberry consequently yields a poor feed conversion ratio (FCR) and substantial resource wastage. In addition, fresh mulberry leaves possess a distinct astringent taste, and high dietary inclusion rates reduce overall feed palatability and voluntary feed intake of animals [5,6]. Therefore, developing efficient strategies to degrade ANFs and upgrade the nutritional profile of mulberry feed resources carries crucial practical value.

Solid-state fermentation (SSF) is capable of degrading ANFs in agricultural by-products and enhancing their nutritional quality via microbial strains and extracellular enzymes, thereby improving feed palatability and in vivo digestibility [7]. As an economical and environmentally benign bioprocess, SSF features low energy consumption and minimal chemical input [8]. Specific microorganisms can synthesize cellulase, hemicellulase and lignolytic enzymes during fermentation. These enzymes depolymerize macromolecular ANFs into low-molecular-weight nutrients, such as oligosaccharides and peptides. These nutrients are more readily absorbable by animals [3,9]. Meanwhile, fermentation reshapes the composition of bioactive metabolites, including polyphenols and flavonoids, and elevates the concentrations of core nutrients such as CP, which further optimizes the feeding performance and nutritional value of forage [10,11,12].

LAB are one of the most commonly applied microbial inoculants for SSF [13]. LAB synthesize lactic and acetic acids to inhibit the proliferation of detrimental microbes, while simultaneously mitigating excessive proteolysis and reducing nutrient losses throughout fermentation [14]. Wu et al. [15] reported consistent observations. Inoculation with *Lactobacillus rhamnosus* markedly reduced silage pH. It also increased lactic acid accumulation. It suppressed pathogenic populations. It restructured the microbial community of alfalfa forage. Even so, LAB are limited by insufficient secretion of hydrolytic enzymes, which constrains their ability to improve substrate digestibility [16].

To address this limitation, the present study implements co-inoculation of LAB and yeast for the SSF of mulberry forage. Yeast strains produce cellulase during fermentation (Cellulases break down cellulose and other high-molecular-weight carbohydrates in plant cell walls, converting them into small molecules including monosaccharides and potential amino acid precursors. These products provide essential carbon and nitrogen sources for microbial fermentation), and the acidic microenvironment generated by LAB facilitates yeast metabolic activity. The synergistic interaction between the two microbial groups collectively improves the overall lignocellulose degradation efficiency of mulberry substrate [17,18,19].

Notably, most existing fermentation studies on forage mulberry have focused on leaf utilization, while the high lignin content in branches, which severely limits whole-plant degradation efficiency, has received little attention [9,20]. Commercial microbial inoculants used for forage fermentation are either single strains (e.g., LAB) or broad-spectrum compound agents, and they lack targeted synergistic effects specifically for lignocellulose degradation in mulberry branches. Although some studies have reported mixed-strain fermentation, the optimization of key parameters such as strain ratio, solid-to-water ratio, and fermentation time for synergistic degradation of lignin and cellulose in whole-plant forage mulberry remains underexplored. This research gap calls for a fermentation strategy tailored to whole-plant forage mulberry. We hypothesized that a mixed inoculum of LBA and yeast could form an acid–enzyme synergistic mechanism. To test this hypothesis, this study had three main objectives: to optimize the SSF process parameters for whole-plant forage mulberry using a mixed inoculum of *L. plantarum* and *P. membranifaciens*; to investigate the dynamic changes in pH and cellulase activity during fermentation and to explore the acid–enzyme synergistic mechanism based on the observed temporal correlations and literature support; and to evaluate the nutritional quality and in vitro digestibility of the fermented forage mulberry, thus providing a technical solution for its use as a non-grain feed in animal nutrition.

## 2. Materials and Methods

### 2.1. Experimental Materials

#### 2.1.1. Forage Mulberry

Two-year-old whole-plant forage mulberry (cv. Guiyou 12) was planted in Village 13, Caoba Town, Mengzi City, Yunnan Province. The whole plant was harvested, mechanically crushed, and dried at 50 °C in an electric thermostatic drying oven (DHG-9070A, Shanghai Jinghong Experimental Equipment Co., Ltd., Shanghai, China), then sealed and stored for later use. (The moisture content of the dried mulberry substrate was not determined in the present study, which may introduce potential variability in the actual substrate moisture during fermentation and further affect the stability and reproducibility of experimental results. Future studies will precisely measure and standardize substrate moisture content prior to fermentation to ensure consistent and controllable experimental conditions.)

#### 2.1.2. Test Strains

*L. plantarum* CF-LJ-4-2 and *P. membranifaciens* CF-B102-1-M were provided by the Institute of Agro-Product Processing, Yunnan Academy of Agricultural Sciences. The strains were preserved in 40% glycerol at −80 °C for long-term storage.

#### 2.1.3. Culture Media

MRS medium (Man, Rogosa, and Sharpe): peptone (Beijing Aoboxing Biotechnology Co., Ltd., Beijing, China) 10 g, beef extract (Beijing Aoboxing Biotechnology Co., Ltd., Beijing, China) 10 g, yeast powder (Angel Yeast Co., Ltd., Yichang, China) 5 g, K_2_HPO_4_ (Sinopharm Chemical Reagent Co., Ltd., Shanghai, China) 2 g, diammonium citrate (Sinopharm Chemical Reagent Co., Ltd., Shanghai, China) 2 g, sodium acetate (Sinopharm Chemical Reagent Co., Ltd., Shanghai, China) 5 g, glucose (Sinopharm Chemical Reagent Co., Ltd., Shanghai, China) 20 g, Tween-80 (Sinopharm Chemical Reagent Co., Ltd., Shanghai, China) 1 mL, MgSO_4_·7H_2_O (Sinopharm Chemical Reagent Co., Ltd., Shanghai, China) 0.58 g, MnSO_4_·4H_2_O (Sinopharm Chemical Reagent Co., Ltd., Shanghai, China) 0.25 g, and agar (Beijing Aoboxing Biotechnology Co., Ltd., Beijing, China) 15 g were dissolved in distilled water, and the final volume was adjusted to 1000 mL. The medium was sterilized at 121 °C (0.1 MPa) for 15 min before use.

YPD medium (Yeast Peptone Dextrose): peptone (Beijing Aoboxing Biotechnology Co., Ltd., Beijing, China) 10 g, yeast powder (Angel Yeast Co., Ltd., Yichang, China) 10 g, glucose (Sinopharm Chemical Reagent Co., Ltd., Shanghai, China) 20 g, and agar (Beijing Aoboxing Biotechnology Co., Ltd., Beijing, China) 15 g were dissolved in distilled water, and the final volume was adjusted to 1000 mL. Sterilization was performed at 121 °C (0.1 MPa) for 15 min.

MRS medium for *L. plantarum* and YPD medium for *P. membranifaciens* were purchased from Beijing Aoboxing Biotechnology Co., Ltd. (Beijing, China). Carboxymethylcellulose sodium (CMC-Na, Sinopharm Chemical Reagent Co., Ltd., Shanghai, China), 3,5-dinitrosalicylic acid (DNS, Aladdin Biochemical Technology Co., Ltd., Shanghai, China), and other analytical reagents (all of analytical grade, from Sinopharm Chemical Reagent Co., Ltd., Shanghai, China) were used as received.

### 2.2. Experimental Design and Methods

#### 2.2.1. Preparation of Seed Solution

*L. plantarum* CF-LJ-4-2: a 0.1 mL aliquot of the glycerol-preserved strain (stored at −80 °C) was inoculated into 100 mL sterile MRS broth (without agar) and cultured at 37 °C with shaking at 180 rpm for 24 h to obtain the seed solution (viable count: 1.2 × 10^9^ CFU/mL).

*P. membranifaciens* CF-B102-1-M: a 0.1 mL aliquot of the glycerol-preserved strain (stored at −80 °C) was inoculated into 100 mL sterile YPD broth (without agar) and cultured at 32 °C with shaking at 180 rpm for 36 h to obtain the seed solution (viable count: 8.5 × 10^8^ CFU/mL).

The two seed cultures were mixed at a volume ratio of 1:1 to prepare the mixed inoculum. The strain combination and mixing ratio were determined based on preliminary experiments optimizing fermentation strains, inoculum concentration, and mixed-strain ratios.

#### 2.2.2. Preparation of Fermentation Substrate

The preserved crushed forage mulberry was rehydrated to a 1:1 solid-to-water ratio and sterilized at 121 °C (0.1 MPa) for 15 min. After cooling to room temperature, 100 g of substrate was placed into sterile fermentation bags with one-way exhaust valves. The mixed inoculum was added and thoroughly mixed. The bags were then sealed for fermentation.

#### 2.2.3. Single-Factor Experiment Design

Using the CF degradation rate as the response variable, single-factor experiments were carried out to examine the effects of four parameters on fermentation efficiency under the following initial conditions: fermentation temperature 32 °C, fermentation time 28 days, inoculum dosage 30%, and solid-to-water ratio 1:1.0. The levels of each factor were set as follows:

Fermentation temperature: 28 °C, 32 °C, 36 °C, 40 °C, 44 °C;

Fermentation time: 7 days, 14 days, 21 days, 28 days, 35 days;

Inoculum dosage (*v*/*w*, based on substrate dry weight): 15%, 20%, 25%, 30%, 35%;

Solid-to-water ratio (*w*/*v*): 1:1.0, 1:1.2, 1:1.4, 1:1.6, 1:1.8.

Each treatment was performed in three replicates. The CF content of the fermented product was measured to calculate the degradation rate.

#### 2.2.4. Orthogonal Experiment and Verification Test Design

Based on the single-factor experiment results, an L_9_ (3^4^) orthogonal array was used to further optimize the fermentation process. Fermentation temperature, fermentation time, inoculum dosage, and solid-to-water ratio were the independent variables, each at three levels (Table 1). A total of nine treatment groups were set up, with three replicates per group (Table 2). The CF degradation rate was used as the evaluation index to select the optimal parameter combination. Verification tests were then carried out under two conditions: the optimal single-factor parameters and the optimal combination from the orthogonal experiment. The CF degradation rates of the two groups were compared to confirm the advantage of the optimized process.

#### 2.2.5. Determination of Fermentation Process Dynamic Indicators

(1) pH dynamic detection: samples were collected at 0, 1, 2, 3, 4, 5, 6, and 7 days. For each sample, 10 g of fermented substrate was mixed with 40 mL of sterile deionized water, shaken at 100 r/min for 20 min at room temperature, and allowed to stand for 10 min. The pH of the supernatant was measured using a precision pH meter (Mettler Toledo, FE28-Standard, Greifensee, Switzerland; accuracy ± 0.01). Each measurement was performed in triplicate.
(2) Cellulase activity dynamic detection: crude enzyme extract was prepared by mixing 10 g of fermented substrate with 40 mL of sterile PBS (pH 7.0), shaking at 150 r/min for 30 min at 4 °C, and centrifuging at 8000× *g* for 15 min. Cellulase activity (filter paper activity, FPA) was measured using the DNS method with filter paper as the substrate. Endoglucanase (CMCase) activity was determined using CMC-Na as the substrate. One unit (U) of cellulase activity was defined as the amount of enzyme required to release 1 μmol of reducing sugar per minute at 50 °C and pH 4.8. A single *P. membranifaciens* control group was set with identical substrate, culture temperature, moisture and inoculation volume (only without *L. plantarum*) to separately quantify the baseline cellulase activity of pure yeast monoculture for subsequent synergistic ratio calculation. The cellulase activities of *L. plantarum* and *P. membranifaciens* were determined separately using selective media (MRS and YPD, respectively). Each sample was analyzed in triplicate.

#### 2.2.6. Comparative Experiment

Forage mulberry was fermented under the final optimized process parameters. A non-fermented control group was prepared under the same substrate conditions but without inoculum addition. The nutritional components of the fermented and control groups were measured and compared to evaluate the effect of fermentation on nutritional quality improvement.

#### 2.2.7. Determination of Nutritional Components

All nutritional component analyses were performed in triplicate. The methods were as follows:

CF was determined according to the national standard GB/T 6434-2022 (filter bag method) [21]. CP was determined by the Kjeldahl method following GB/T 6432-2018 [22]. EE was determined by the Soxhlet extraction method according to GB/T 6433-2025 [23]. Crude ash (CA) was determined by the muffle furnace incineration method following GB/T 6438-2025 [24]. Carbohydrates were calculated by difference (100% − CP% − EE% − Ash% − CF%) with reference to the method described by [25]. Amino acids (AAs) contents were determined using a fully automatic amino acid analyzer (e.g., Hitachi L-8900, Tokyo, Japan) according to the method specified in GB/T 18246-2019 [26].

#### 2.2.8. In Vitro Simulated Rumen Digestion Experiment

A commercial standardized rumen microbial inoculum was adopted to replace fresh rumen fluid collected from fistulated ruminants. The inoculant used in this experiment was rumen-specific high-activity yeast composite preparation (Cat. No. AQ-RM01), which was purchased from Angel Yeast Co., Ltd. (Yichang, China). Before use, the inoculant was stored at 4 °C under sealed dark conditions. Artificial saliva was prepared following the classic formula, and mixed evenly with the commercial rumen inoculum at a fixed proportion under strict anaerobic environment. The configuration, incubation temperature, substrate-to-inoculant ratio and culture duration of the whole in vitro fermentation system were operated uniformly in accordance with the manufacturer’s standard operating instructions for all treatment groups.

All tests carried out in this research were purely in vitro simulation experiments. Commercial standardized rumen microbial inoculant was used to substitute rumen fluid derived from live ruminants. No live animal feeding, tissue sampling or any invasive animal operations were implemented throughout the whole experiment. Hence, animal ethics approval was not required for this study.

#### 2.2.9. Data Processing and Analysis

Microsoft Excel 2019 (version 2408, Microsoft Corporation, Redmond, WA, USA) was used for data organization, and SPSS Statistics 26.0 (IBM Corporation, Armonk, NY, USA) was used for statistical analysis. Duncan’s multiple range test was used for multiple comparisons of means. All results were expressed as mean ± standard deviation (SD). Differences were considered statistically significant at *p* < 0.05 and highly significant at *p* < 0.01.

## 3. Results and Analysis

### 3.1. Results of Single—Factor Experiment for Optimizing Fermentation Process Parameters of Forage Mulberry

#### 3.1.1. Effect of Fermentation Temperature on CF Degradation Rate of Forage Mulberry

**Figure 1:**
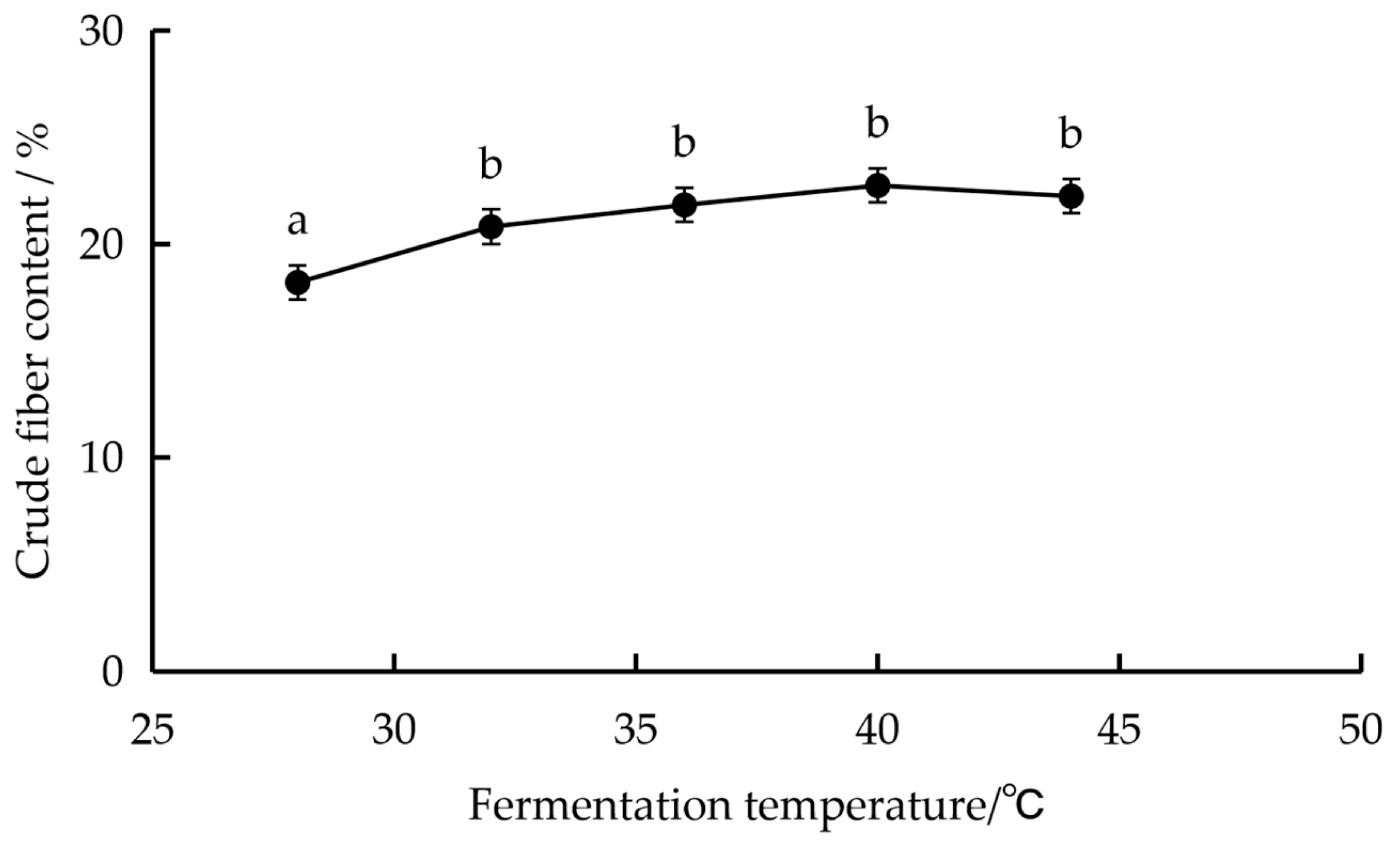
Effect of fermentation temperature on the CF degradation rate of forage mulberry. (Note: Different lowercase letters indicate significant differences among groups (*p* < 0.05)).

As shown in Figure 1, the CF content in the dry matter of feed mulberry exhibited an initial increase followed by a decrease with rising fermentation temperature. At 28 °C, the CF content reached its lowest value (18.20%), which was significantly lower than those at the other tested temperatures (*p* < 0.05). When the temperature increased to 40 °C, the CF content gradually rose to a peak of 22.74%, then slightly decreased to 22.24% at 44 °C. The CF content at 28 °C was significantly different from the other temperatures (32–44 °C, *p* < 0.05). No significant differences were found among the four higher temperatures (*p* < 0.05).

This trend may be attributed to the active growth, reproduction, and metabolism of the strain at 28 °C, which secrete substantial amounts of cellulase, hemicellulase, and other lignocellulose-degrading enzymes, thereby effectively decomposing the CF and lignocellulosic complexes in mulberry leaves. And when the fermentation temperature exceeds 32 °C, it approaches the upper limit of the strains’ thermal tolerance, inhibiting cell proliferation and reducing the synthesis and catalytic activity of key degrading enzymes, thus impairing CF degradation. At 44 °C, although the further temperature increase exacerbates the inhibition of strain activity, the slight decrease in CF content may be associated with the accumulation of acidic metabolites (e.g., lactic acid) in the early stage, which moderately promotes the dissolution of some hemicellulose components.

These results indicate that 28 °C is the optimal fermentation temperature for forage mulberry.

#### 3.1.2. Effect of Fermentation Time on CF Degradation Rate of Forage Mulberry

**Figure 2:**
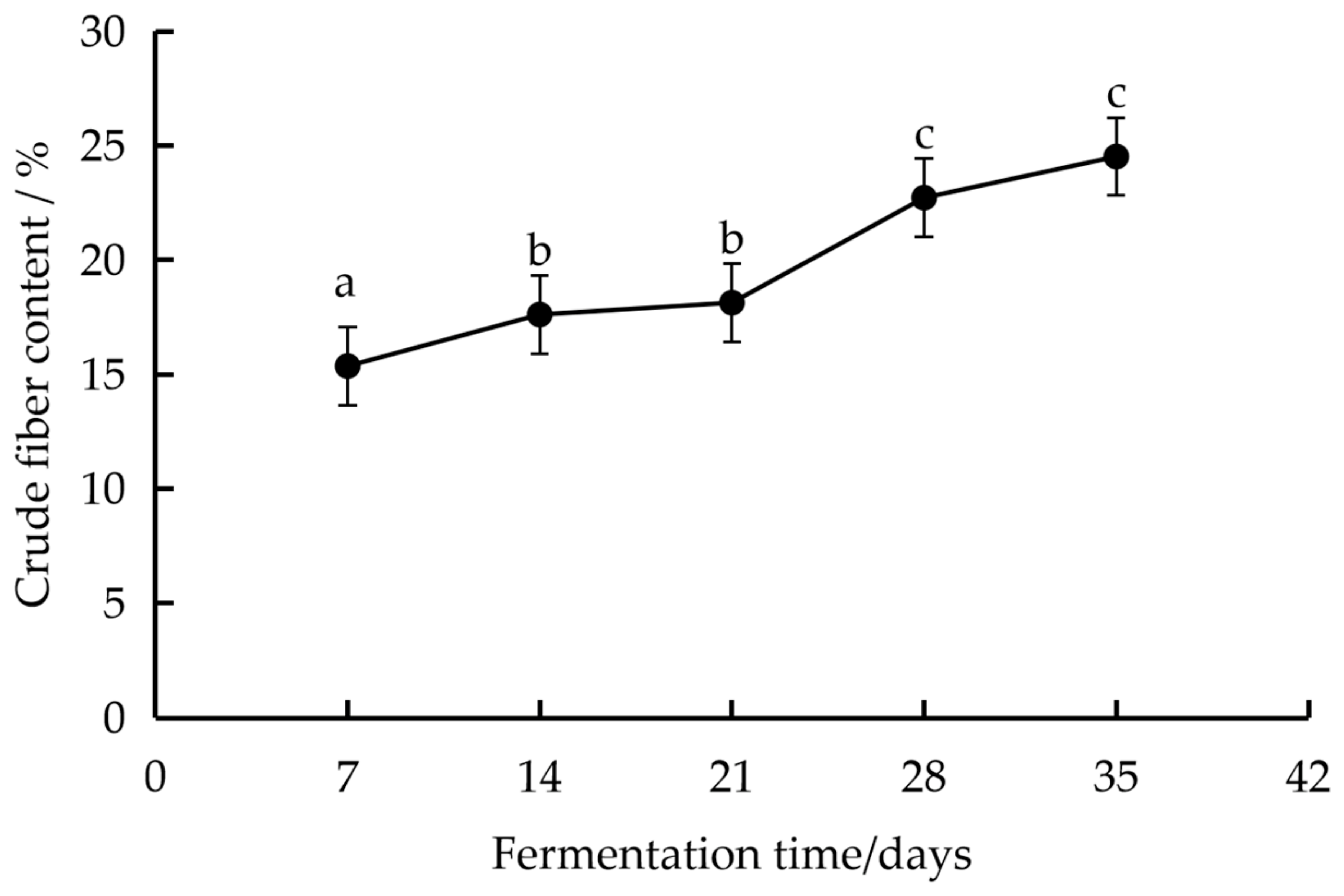
Effect of fermentation time on the CF degradation rate of forage mulberry. (Note: Different lowercase letters indicate significant differences among groups (*p* < 0.05)).

As shown in Figure 2, the CF content of forage mulberry increased continuously with longer fermentation time. At 7 days of fermentation, the CF content reached its minimum (15.36%). This value was significantly lower than at 14, 21, 28, and 35 days (*p* < 0.05). When fermentation was extended to 14 and 21 days, the CF content increased slowly. It reached 17.61% and 18.13%, respectively. Further extension to 28 and 35 days led to a rapid increase. The CF content reached 22.72% and 24.52%, respectively. Statistical analysis showed no significant differences between 14 and 21 days (*p* > 0.05). The same was true between 28 and 35 days (*p* > 0.05).

This trend may be attributable to the early stage (day 7). During this stage, the strain entered the logarithmic growth phase. Abundant substrates were available (e.g., soluble carbohydrates). The microenvironment was also favorable (neutral pH). Under these conditions, the strain exhibited vigorous metabolic activity. It secreted substantial amounts of cellulase, hemicellulase, and other lignocellulose-degrading enzymes. These enzymes efficiently decomposed the CF. This resulted in the lowest CF content. As fermentation progressed, substrates were gradually consumed. Acidic metabolites (e.g., lactic acid) accumulated. This lowered the fermentation pH to about 4.0–4.5. The lower pH inhibited strain proliferation. It also reduced the synthesis and catalytic activity of degrading enzymes. Consequently, the CF degradation rate slowed down significantly. In addition, prolonged fermentation (over 21 days) induced partial strain autolysis. This released undegraded cell wall components (e.g., residual lignin) into the substrate. These components further contributed to the increase in CF content.

These results indicate that 7 days is the optimal fermentation time for forage mulberry.

#### 3.1.3. Effect of Solid-to-Water Ratio on CF Degradation Rate of Forage Mulberry

**Figure 3:**
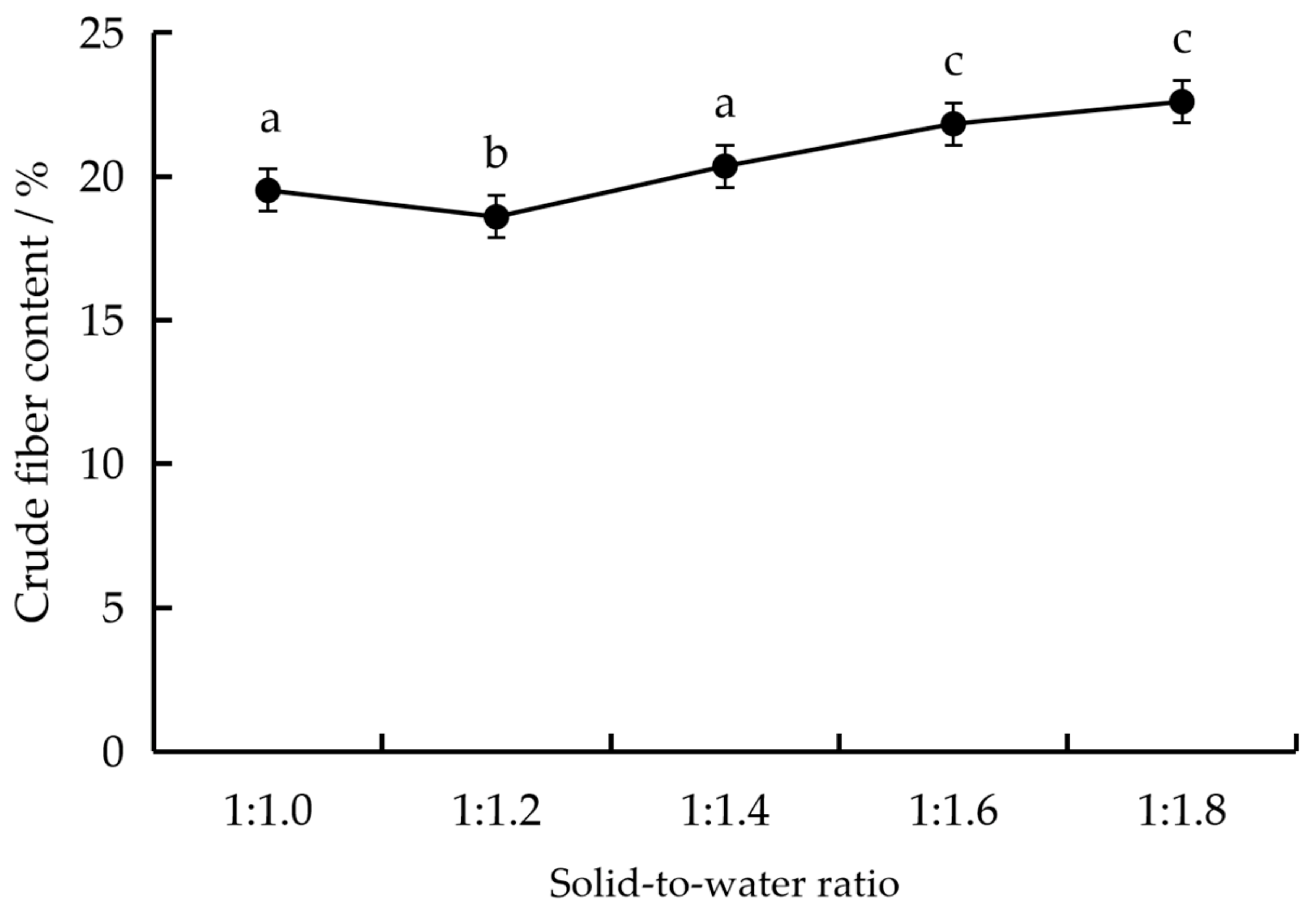
Effect of solid-to-water ratio on the CF degradation rate of forage mulberry. (Note: Different lowercase letters indicate significant differences among groups (*p* < 0.05)).

As shown in Figure 3, the CF content of fermented forage mulberry first decreased and then increased as the solid-to-water ratio rose from 1:1.0 to 1:1.8. At a ratio of 1:1.2, the CF content reached its minimum (18.59%). This value was significantly lower than in all other groups (*p* < 0.05). At 1:1.0, the CF content was 19.51%. When the ratio was increased to 1:1.4, 1:1.6, and 1:1.8, the CF content gradually rose to 20.36%, 21.83%, and 22.60%, respectively. Statistical analysis showed no significant differences between the 1:1.6 and 1:1.8 groups (*p* > 0.05). The same was true between the 1:1.0 and 1:1.4 groups (*p* > 0.05).

This phenomenon may be attributed to the requirement of the mixed strains (*L. plantarum* CF-LJ-4-2 and *P. membranifaciens* CF-B102-1-M) for a balanced moisture environment to sustain metabolic activity. Excessive dryness restricts substrate-strain contact and enzyme diffusion. Excessive wetness reduces oxygen availability, which is detrimental to yeast aerobic metabolism. It also disrupts the carbon-to-nitrogen (C/N) balance. At a solid-to-liquid ratio of 1:1.2, the fermentation system maintained an optimal moisture content. This facilitated microbial adhesion to the substrate surface. It also promoted enzyme secretion and diffusion. In addition, it ensured an adequate nitrogen supply for enzyme synthesis and strain proliferation. This combination achieved the highest CF degradation efficiency. At a low solid-to-water ratio (1:1.0), the substrate was relatively dry. This led to insufficient contact between strains and CF components. It also limited the diffusion of substrates and enzymes. These factors restricted strain growth and cellulase secretion. As a result, CF degradation was incomplete. At high solid-to-water ratios (≥1:1.4), excessive moisture reduced substrate porosity. This caused anaerobic stress for *P. membranifaciens* (a facultative anaerobe) and inhibited its cellulase secretion. In addition, higher water content diluted nitrogen sources. This disrupted the C/N balance and further suppressed lignocellulolytic enzyme synthesis. Thus, CF degradation efficiency decreased.

These results indicate that a solid-to-water ratio of 1:1.2 is optimal for forage mulberry fermentation.

#### 3.1.4. Effect of Inoculum Dosage on CF Degradation Rate of Forage Mulberry

**Figure 4:**
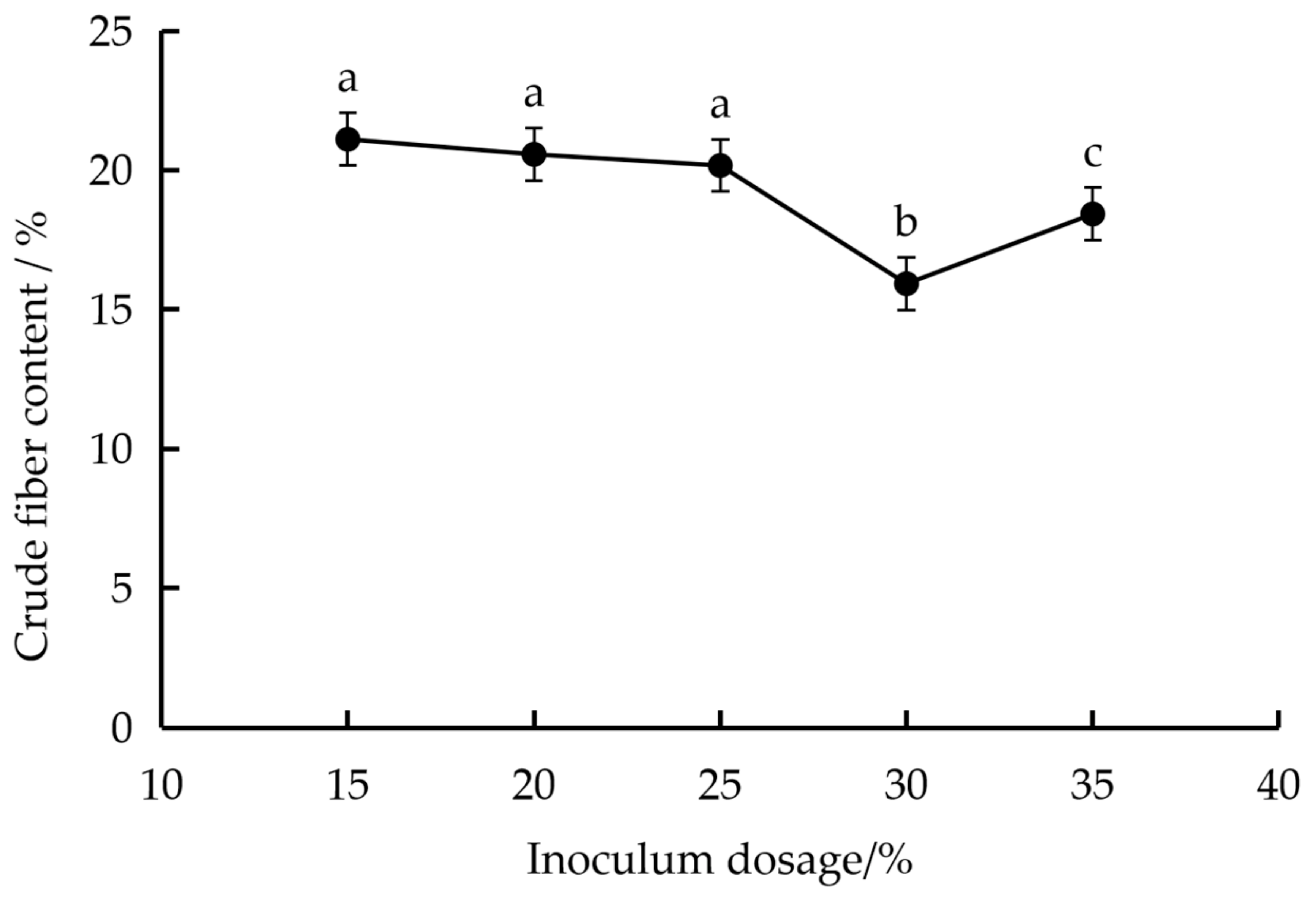
Effect of inoculum dosage on the CF degradation rate of forage mulberry. (Note: Different lowercase letters indicate significant differences among groups (*p* < 0.05)).

As shown in Figure 4, with the inoculation amount increasing from 15% to 35%, the CF content of fermented mulberry branches initially remained stable, then decreased significantly, and subsequently increased slightly. When the inoculation amount ranged from 15% to 25%, the CF content remained above 20%. At an inoculation amount of 30%, the CF content decreased significantly to its lowest value (15.93%). However, when the inoculation amount was further increased to 35%, the CF content slightly rebounded to 18.43%. Statistical analysis showed that the CF content at 30% inoculum dosage was significantly lower than in all other groups (*p* < 0.05). No significant differences were found among the 15%, 20%, and 25% groups (*p* > 0.05). The 35% group had significantly higher CF content than the 30% group, but significantly lower than the 15–25% groups (*p* < 0.05).

This trend may be attributed to the direct influence of inoculation amount on three factors. These are the colonization rate of the mixed strains (*L. plantarum* CF-LJ-4-2 and *P. membranifaciens* CF-B102-1-M), their interspecific synergy, and the stability of the microenvironment. At low inoculation levels (15–25%), the initial cell density was insufficient. This resulted in slow colonization and inadequate cellulase secretion. These factors led to incomplete degradation of CF. Consequently, the CF content remained relatively high. No significant differences were observed among samples within this range. At the optimal level (30%), the strains rapidly adapted to the fermentation environment. They promptly entered the logarithmic growth phase. This maximized their synergistic metabolic interactions. Both strains secreted high levels of cellulase. These enzymes efficiently decomposed the CF. This significantly reduced its content. However, at an excessive inoculation level (35%), the initial cell density was too high. This triggered intense competition for limited substrates (e.g., soluble carbohydrates) and space. At the same time, metabolic by-products (e.g., lactic acid and ethanol) accumulated rapidly. This caused a sharp decline in pH. The low pH in turn inhibited the growth and metabolic activity of both strains. As a result, enzyme synthesis and catalytic activity were impaired. This led to reduced CF degradation and a slight increase in its content.

These results indicate that 30% is the optimal inoculum dosage for forage mulberry fermentation.

### 3.2. Results of Orthogonal Experiment for Optimizing Fermentation Process Parameters of Forage Mulberry

#### 3.2.1. Range Analysis of Orthogonal Experiment

As shown in Table 3, the influence of each fermentation factor on the CF degradation rate was evaluated by intuitive range analysis based on the range (R) values. The R value reflects how much the response index (CF degradation rate) varies with changes in factor levels. A larger R value indicates a stronger influence of that factor.

The four factors were ranked by their influence on the CF degradation rate as follows: fermentation time (A) > fermentation temperature (B) > inoculum dosage (D) > solid-to-water ratio (C). The average values (K values) of the CF degradation rate were calculated for each level of each factor. A higher K value indicated better performance of that level. Based on this, the optimal combination for maximizing the CF degradation rate was A_1_B_2_C_1_D_1_. The factor codes and corresponding parameters are A (fermentation time) = 7 days, B (fermentation temperature) = 32 °C, C (solid-to-water ratio) = 1:1.0, and D (inoculum dosage) = 25%.

#### 3.2.2. Variance Analysis of Orthogonal Experiment

To verify the statistical significance of the factor effects, analysis of variance (ANOVA) was performed on the orthogonal experiment data (Table 4). The corrected ANOVA model for CF degradation rate was highly significant (*p* < 0.01). This confirms the reliability of the orthogonal experimental design. It also indicates no significant interference from random errors.

All four factors significantly affected the CF degradation rate (*p* < 0.05 or *p* < 0.01). These factors were fermentation time (A), fermentation temperature (B), solid-to-water ratio (C), and inoculum dosage (D). In ANOVA, the F-value reflects the relative importance of each factor. A larger F-value means a stronger influence on the response index. Consistent with the range analysis, the order of influence based on F-values was: fermentation time (A) > fermentation temperature (B) > inoculum dosage (D) > solid-to-water ratio (C). The agreement between range analysis and ANOVA supports the reliability of the experimental results. It also provides a solid basis for subsequent parameter optimization and verification.

### 3.3. Results of Verification Experiment

Based on the range analysis and ANOVA, the optimal combination was identified. It was A1B2C1D1. Here, A is fermentation time, B is temperature, C is solid-to-water ratio, and D is inoculum dosage. The corresponding parameters were: 7 days, 32 °C, 1:1.0 solid-to-water ratio, and 25% inoculum. This combination was not among the tested groups in the orthogonal array. It also differed from the optimal parameters from the single-factor experiments. The single-factor conditions were: 7 days, 28 °C, 30% inoculum, and 1:1.2 solid-to-water ratio. Therefore, verification experiments were carried out. They compared these two parameter sets.

The verification results showed that the CF content under the single-factor optimal parameters was 14.12%. This value was significantly lower than that obtained with the orthogonal-predicted combination (16.58%) (*p* < 0.05). This discrepancy may be due to the interactive effects among the four factors in the orthogonal experiment. The limited level settings of the L_9_ (3^4^) array may have restricted the ability to capture the true synergistic optimum. In contrast, single-factor experiments systematically evaluated the independent effect of each parameter, allowing each factor to be optimized for its maximum contribution to CF degradation. Consequently, the optimal fermentation process for forage mulberry was determined as follows: fermentation time 7 days, fermentation temperature 28 °C, inoculum dosage 30%, and solid-to-water ratio 1:1.2.

### 3.4. Results of Comparison Experiment

As shown in Table 5, significant differences in macronutrient components were observed between the fermented forage mulberry (under the optimized process) and the unfermented control group (*p* < 0.05 or *p* < 0.01).

The CF content in the fermented group was significantly lower than in the control group (*p* < 0.01). It decreased from 26.12% to 14.47%. This represents a reduction of 44.60%. The carbohydrate content also decreased significantly (*p* < 0.01). It dropped from 62.62% to 47.25%. This represents a reduction of 32.53%. This may be attributed to the degradation of macromolecular carbohydrates (e.g., cellulose and hemicellulose). The mixed strains (*L. plantarum* and *P. membranifaciens*) secreted lignocellulolytic enzymes. These enzymes converted macromolecular carbohydrates into small-molecule metabolites, such as oligosaccharides and organic acids.

The CP content in the fermented group was significantly higher than in the control group (*p* < 0.01). It increased from 20.66% to 28.50%. This represents a 35.77% increase. This improvement may be attributed to two factors. The first is microbial protein synthesis. During strain proliferation, non-protein nitrogen and carbohydrates were converted into microbial biomass protein [18]. The second is a concentration effect. Fiber degradation caused dry matter loss, which relatively concentrated the protein content. The CA content also increased significantly (*p* < 0.01). It rose from 7.50% to 8.80%. This represents a 17.33% increase. This indicates the enrichment of mineral elements (e.g., calcium and phosphorus) during fermentation. In addition, the EE content was significantly higher in the fermented group (*p* < 0.05). It increased from 9.15% to 15.45%. This represents a 68.85% increase. This may be related to the synthesis of microbial lipids by *P. membranifaciens* under favorable metabolic conditions.

As shown in Table 6, each amino acid in fermented forage mulberry was improved to varying degrees compared with the unfermented control. The contents of TAA, total essential amino acids (TEAA), and total non-essential amino acids (TNEAA) were all significantly higher in the fermented group (*p* < 0.01), further confirming the nutritional improvement effect of the optimized fermentation process.

Among essential amino acids (EAAs), five amino acids showed a significant increase (*p* < 0.01). These were threonine (Thr), valine (Val), isoleucine (Ile), leucine (Leu), and phenylalanine (Phe). Four other EAAs also increased significantly (*p* < 0.05). These were methionine (Met), histidine (His), lysine (Lys), and arginine (Arg). This enhancement may be attributed to two synergistic effects. The first is proteolysis. The mixed strains secreted proteases to hydrolyze the macromolecular proteins in forage mulberry into small peptides and free amino acids. The second is microbial synthesis. *L. plantarum* synthesized and accumulated several EAAs. These EAAs cannot be synthesized by monogastric animals. This process increased the overall EAA content.

Several NEAAs increased significantly (*p* < 0.01): Asp, Ser, Glu, Gly, Ala, Tyr, and Pro. Only Cys remained unchanged. Glu and Asp showed the largest increases. They are related to palatability and may improve livestock feed intake. The stable Cys level may be due to its use in glutathione synthesis, preventing its accumulation. The TEAA/TAA ratio decreased after fermentation. Although each EAA increased in absolute terms, NEAAs (especially flavor amino acids like Glu and Asp) accumulated faster. This reduced the relative EAA proportion in TAA. The TEAA/TNEAA ratio also declined, suggesting preferential synthesis of NEAAs by microbial metabolism during SSF.

### 3.5. Dynamic Changes During Fermentation

#### 3.5.1. pH

**Figure 5:**
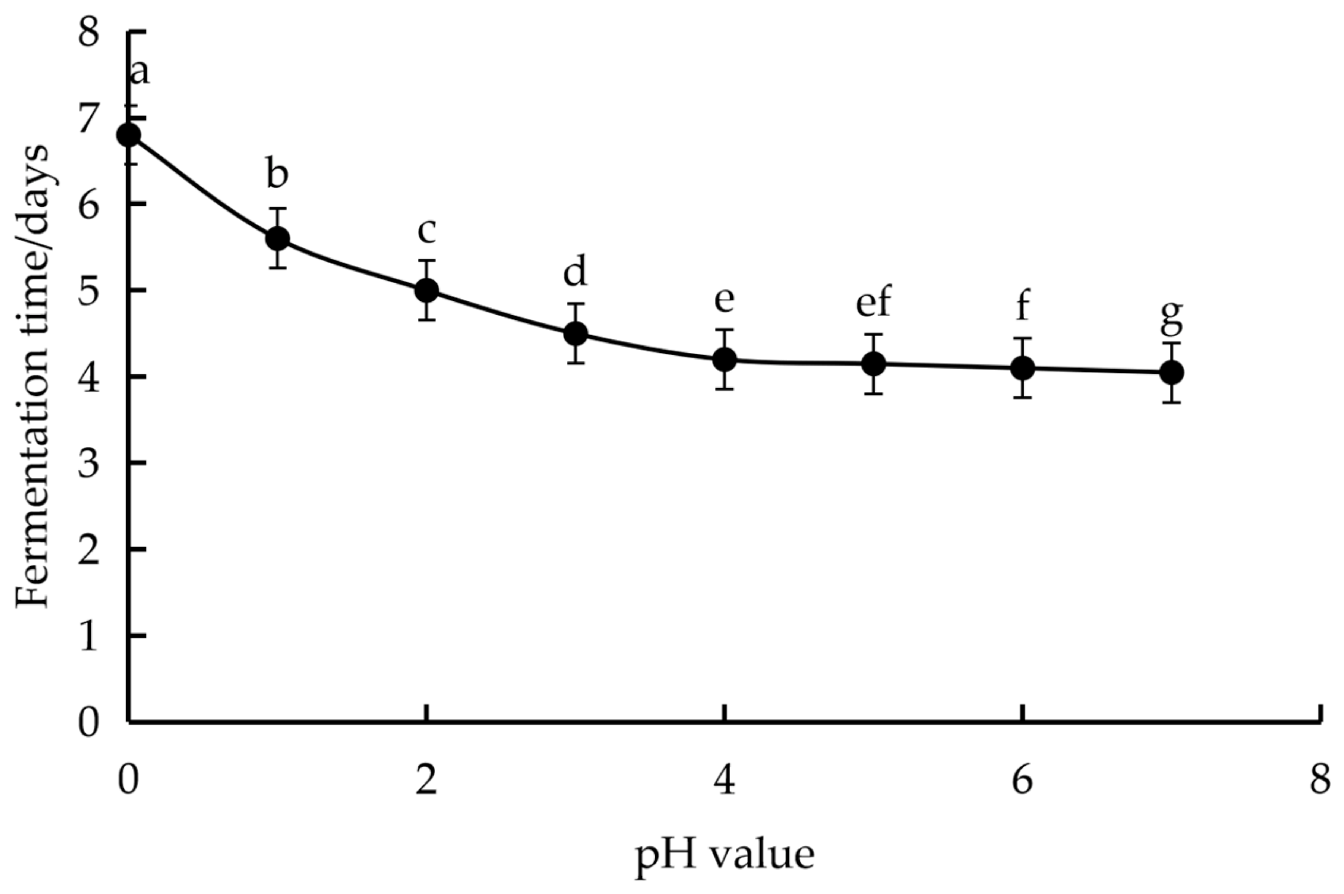
Dynamic changes in pH during fermentation. (Note: Different lowercase letters indicate significant differences among groups (*p* < 0.05)).

As shown in Figure 5, the pH of the fermentation system showed a rapid decrease followed by a stable trend. The initial pH of the unfermented substrate was 6.8. During the first day, the pH dropped rapidly to 5.0, which was likely attributed to lactic acid synthesized by *L. plantarum*. From day 1 to day 2, the pH continued to decrease to 4.5 and then remained stable at 4.0–4.5 until the end of fermentation (day 6). This weakly acidic environment may be conducive to the optimal pH range for cellulase activity (4.0–5.0), while possibly suppressing pathogenic microbes, which might together form a pH-enzyme synergistic fermentation system.

#### 3.5.2. Cellulase Activity

**Figure 6:**
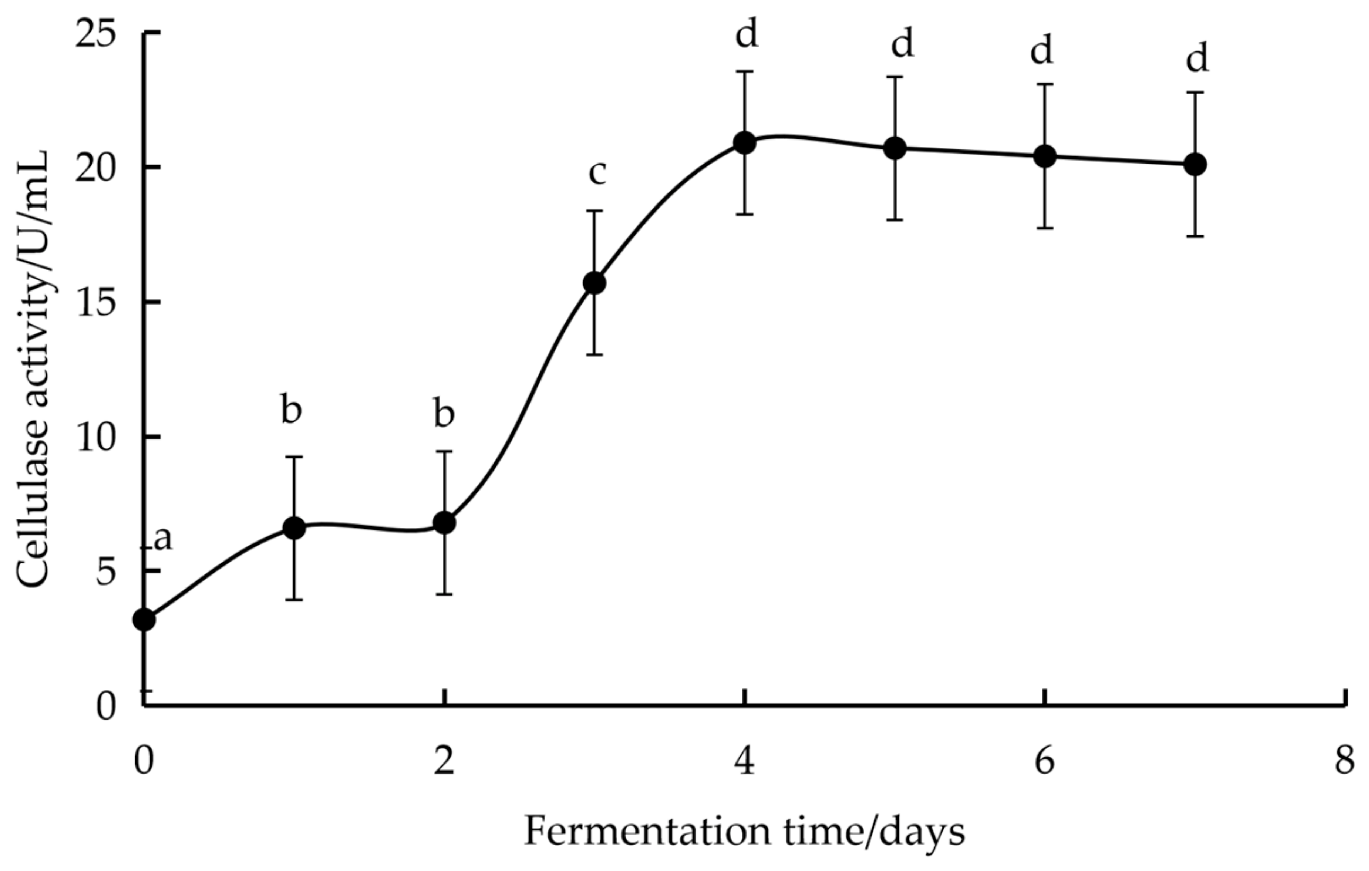
Dynamic changes in cellulase activity during fermentation. (Note: Different lowercase letters indicate significant differences among groups (*p* < 0.05)).

As shown in Figure 6, the cellulase activity during fermentation showed a slow increase, then a rapid rise, and finally a stable plateau. In the early stage (0–1 day), the system pH was relatively high. This may have resulted in weak enzyme production and a gentle increase in cellulase activity. As LAB produced organic acids, the system pH gradually dropped to a weakly acidic level. Cellulase activity then increased rapidly after 2 days and reached its maximum (20.90 U/mL) at 3 days. From day 3 to the end of fermentation, enzyme activity decreased slightly and remained stable.

Correlation analysis suggested an extremely significant negative correlation between system pH and total cellulase activity. This indicates that the weakly acidic environment may have effectively enhanced the overall enzyme production capacity of the mixed strains. It may also have contributed to a pH-enzyme synergistic effect.

Under identical culture conditions, the peak cellulase activity of single *P. membranifaciens* monoculture was 13.86 U/mL. Compared with the single yeast fermentation group, the maximum cellulase activity of the co-fermentation system was increased by 50.84%, this suggests a potential synergistic interaction between LAB and yeast.

### 3.6. Characteristics of In Vitro Simulated Rumen Digestion

As shown in Table 7, the in vitro DMD and CP digestibility of fermented forage mulberry were 72.30% and 78.60%, respectively. These values were significantly higher than those of the unfermented control (*p* < 0.01). This improved digestibility may be closely associated with two factors. One is the degradation of lignocellulose. The other is the accumulation of soluble nutrients. These changes may weaken the physical barrier that hinders digestion by rumen microorganisms.

## 4. Discussion

### 4.1. Effects of Fermentation Process on Nutritional Composition of Feed Mulberry

Changes in nutritional composition are important indicators for evaluating the quality of fermented feed. Among these, cellulose content serves as a core parameter for assessing nutritional value. It significantly influences both digestibility and feed intake in animals. The lower the cellulose content, the better the feed quality. In this study, a solid-state fermentation (SSF) process was adopted. The CF degradation rate was used as the key performance indicator. The SSF process for feed mulberry was optimized through single-factor experiments and an L9(3^4^) orthogonal array design. The optimal parameters were determined as follows: fermentation time of 7 days, fermentation temperature of 28 °C, inoculum amount of 30%, and solid-to-liquid ratio of 1:1.2. Under these conditions, the CF degradation rate reached 44.60%, and the CP content increased by 35.77%. Gao [27] performed a similar study on mulberry leaves. He first used Aspergillus niger for aerobic fermentation. Then, he applied Saccharomyces cerevisiae and *L. plantarum* for anaerobic fermentation. The SSF process was optimized through single-factor and orthogonal experiments. The final results showed that CP increased by 51.97% and CF degraded by 56.12% compared with unfermented mulberry leaves. These values were significantly higher than those obtained in the present study. This discrepancy may be attributed to two factors. First, the process in this study was relatively simple. Second, whole-plant feed mulberry (including stems) was used for fermentation. The lignin in mulberry branches was difficult to degrade sufficiently. Qiao et al. [28] reviewed the research progress of probiotic fermentation processes. They confirmed that SSF is more suitable for high-fiber raw materials such as straw and mulberry branches/leaves. SSF has a nutrient retention rate 12–15% higher than liquid-state fermentation. Therefore, SSF was selected to optimize the fermentation process in the current study. Notably, the validation experiments in this study showed that the single-factor optimal process (CF content of 14.12%) outperformed the orthogonal combination (CF content of 16.58%). This discrepancy may be due to two reasons. One reason is that the factor levels in the orthogonal design did not fully cover the optimal ranges identified in the single-factor experiments. Another is that complex synergistic interactions may exist among factors. In contrast, single-factor experiments more intuitively reflect the effect of individual variables on fermentation performance. In addition, the pretreatment process adopted in this study included sterilization at 121 °C for 15 min. This step not only reduced the risk of microbial contamination but also disrupted the cell wall structure of feed mulberry.

The orthogonal array experiment and the single-factor verification test gave different results. This discrepancy may be due to the L_9_ (3^4^) design. This design uses only three discrete levels per factor. As a result, it cannot fully capture the continuous response relationships between CF degradation and individual variables. In addition, the orthogonal design does not account for potential interactions between adjacent factor levels. This is especially true when the response surface is nonlinear. In contrast, single-factor experiments allow a more detailed exploration of each factor. They cover a broader range (five gradient levels per factor). They do this without mutual interference from other variables. Therefore, they provide a more direct and accurate identification of the optimal level for each factor.

The orthogonal design revealed the order of influence of the four factors. The order was time > temperature > inoculum dosage > solid-to-water ratio. This served as a useful reference for parameter screening. However, the verification test directly confirmed that the single-factor combined scheme gave a lower CF content (14.12%) than the orthogonal prediction (16.58%). Therefore, the final fermentation conditions were selected based on the single-factor verification result. This result was experimentally validated to be superior to the orthogonal combination. This also suggests that orthogonal designs with limited levels may not always identify the global optimum. Future studies could adopt response surface methodology to further refine the optimization.

### 4.2. Acid–Enzyme Synergistic Mechanism

The selection of fermentation additives directly influences the nutritional conversion efficiency of feed mulberry. Previous studies have demonstrated a synergistic effect. The appropriate combination of microbial inoculants, enzyme preparations, and compound additives can achieve this effect. It is often described as “1 + 1 > 2” [29]. Compound microbial inoculants (e.g., *L. plantarum* and *Bacillus spp*.) can competitively inhibit detrimental microorganisms (e.g., *Escherichia coli*). They also promote lactic acid production by LAB. This reduces environmental pH. The lower pH activates endogenous cellulase. This enzyme then decomposes hemicellulose. Finally, this process releases CP and water-soluble carbohydrates. These nutrients were originally entrapped by the cell wall [30]. Compound enzyme preparations (e.g., cellulase and xylanase) can specifically hydrolyze the β-1,4-glycosidic bonds in plant cell walls. This disrupts the three-dimensional network structure composed of cellulose, hemicellulose, and lignin. Among these enzymes, xylanase effectively degrades arabinan. It also significantly reduces digesta viscosity. This eliminates the physical encapsulation of nutrients such as starch and protein. As a result, CP solubility increases by 12–18% [31].

In the present experiment, fermentation with a compound microbial inoculant was compared with unfermented feed mulberry. The inoculant contained *L. plantarum* CF-LJ-4-2 and *P. membranifaciens* CF-B102-1-M at a 1:1 ratio. After fermentation, CP increased by 35.77%, TAA by 57.31%, and EE by 68.85%. CF and carbohydrate contents decreased by 44.60% and 32.53%, respectively. The degradation efficiency was significantly superior to most comparable studies. For example, Ding et al. [32] used a combination of *L. plantarum* and *Bacillus subtilis*. They achieved a maximum cellulose degradation rate of only 5.01%. This is far lower than the 44.60% obtained in the present study. The core difference between the two strain combinations may lie in the superior metabolic synergy of “*L. plantarum* + *P. membranifaciens*” used in this study. Under the fermentation conditions established in this study (28 °C), *L. plantarum* may rapidly metabolize and produce lactic acid, which could maintain the fermentation system at a weakly acidic pH of 4.0–4.5. This weakly acidic environment may inhibit the growth of contaminating microorganisms and might contribute to the improved cellulase secretion activity of *P. membranifaciens* (presumably associated with the activation of acid-sensing proteins on the yeast cell wall). This reciprocal interaction established a virtuous cycle. The cycle was “acid production-enzyme promotion-synergistic degradation”. This ultimately achieved efficient decomposition of the recalcitrant lignin-cellulose complex in mulberry branches. Duan et al. [6] fermented feed mulberry with different concentrations of *L. plantarum* CF-LJ-4-2 and *P. membranifaciens* CF-B102-1-M, respectively. Their results showed the following. When the inoculation concentration of *L. plantarum* CF-LJ-4-2 ranged from 5% to 25%, CP content increased by up to 2.42%. TAA increased by up to 11.02%. EE increased by up to 119.84%, with the maximum post-fermentation EE content being only 5.54%. CF degradation reached up to 24.01%. When the inoculation concentration of *P. membranifaciens* CF-B102-1-M ranged from 5% to 25%, CP content increased by up to 8.54%. TAA increased by up to 8.89%. EE increased by up to 15.08%. CF degradation reached up to 19.23%. Hu et al. [33] investigated the effects of *Lactobacillus acidophilus* on the fermentation quality of feed mulberry. Compared with the control group, CP content peaked at day 7 of fermentation. It increased from 13.62% to 13.83% (a 1.54% increase). The best fiber degradation was observed at day 4. Neutral detergent fiber decreased from 36.50% to 30.69% (an 18.93% reduction). Acid detergent fiber decreased from 26.06% to 21.47% (a 21.38% reduction). Deng [34] fermented feed mulberry with *L. plantarum* CAU-a214. He reported a 6.67% increase in CP content. He also reported reductions of 18.86% in neutral detergent fiber and 12.90% in acid detergent fiber. Collectively, these findings indicate that single-strain fermentation was considerably less effective than the mixed-strain fermentation used in the present study.

### 4.3. Nutritional Quality and In Vitro Digestibility

The significant reduction in CF content after fermentation may be attributed to the synergistic degradation of lignocellulose. Cellulase, hemicellulase, and ligninase secreted by the mixed strains contributed to this degradation [35]. The increase in CP content may be due to two factors. One is microbial protein synthesis. The other is the release of bound proteins from the lignocellulosic matrix [36]. The enrichment of TAA, especially essential amino acids, improves the nutritional balance of forage mulberry. This makes it more suitable for livestock requirements. The enhanced in vitro DMD and CP digestibility further confirm the practical application potential of fermented forage mulberry in animal nutrition.

CA and EE also increased significantly after fermentation. The rise may be mainly caused by the breakdown of lignocellulosic macromolecules, which possibly releases mineral elements wrapped in fiber structures, coupled with inorganic metabolites produced during microbial metabolism. The elevated EE may originate from lipid biosynthesis of *P. membranifaciens* under the suitable weak acidic microenvironment formed during fermentation.

Although the absolute contents of all essential amino acids were significantly improved after fermentation, the TEAA/TAA and TEAA/TNEAA ratios decreased markedly. This phenomenon may be explained by the preferential synthesis of non-essential flavor amino acids, such as glutamic acid and aspartic acid, by the mixed strains through proteolysis and de novo biosynthesis. The accumulation rate of these non-essential amino acids could be higher than that of essential amino acids, which might reduce the relative proportion of essential amino acids in TAA. Alternatively, this proportional decline may simply reflect the substantial increase in non-essential amino acid content rather than a true reduction in essential amino acid quality. Further studies are needed to clarify the amino acid metabolic flux of each strain during fermentation.

In vitro ruminal fermentation technology is widely recognized as a classic high-throughput screening method. It can rapidly evaluate the nutritional improvement effect of fermented forage. This approach avoids the high cost and complicated animal ethical procedures of in vivo feeding trials. The core objective of this study was twofold. First, we optimized the solid-state mixed fermentation parameters of whole-plant forage mulberry. Second, we aimed to clarify the synergistic mechanism between LAB and yeast. This mechanism is mediated by pH-cellulase interaction. The in vitro digestibility index served as a comprehensive auxiliary indicator. It was used to quantify the improvement of mulberry feed quality after fermentation. Admittedly, differences exist between in vitro simulation results and real in vivo ruminal metabolic characteristics. Therefore, further in vivo animal feeding experiments will be carried out in follow-up research. These experiments will verify the practical feeding value of fermented mulberry forage.

### 4.4. Application Potential, Limitations, and Future Perspectives

The optimized SSF process has several advantages for industrial application. It uses whole-plant forage mulberry as the substrate. This material is low-cost and widely available. The mixed strains are safe and easy to cultivate. The process requires no complex equipment. This reduces production costs. The fermented product has high nutritional value and good digestibility. Therefore, it is suitable as a non-grain feed for ruminants and monogastric animals.

This study has demonstrated the effectiveness of the SSF process in improving the nutritional quality of forage mulberry. However, some analyses were beyond the scope of this work. These include molecular-level analyses, such as enzyme-related gene expression and microbial community dynamics during fermentation. This study focused primarily on process parameter optimization and nutritional quality evaluation.

In addition, the experimental design (Section 2.1.1) the moisture content of the dried mulberry substrate was not determined in the present study, which may introduce potential variability in the actual substrate moisture during fermentation and further affect the stability and reproducibility of experimental results. Future studies will precisely measure and standardize substrate moisture content prior to fermentation to ensure consistent and controllable experimental conditions. (Section 2.2.5 (2)) included only a yeast-only monoculture control, without a corresponding *L. plantarum* only control group. Consequently, the individual contributions of *L. plantarum* to fiber degradation, pH dynamics, and nutrient changes cannot be quantitatively distinguished from the synergistic effects of the mixed culture. This limitation precludes a full mechanistic attribution of the observed improvements to either strain alone. Nevertheless, the yeast-only control did allow for the quantification of baseline cellulase activity from *P. membranifaciens* and facilitated the calculation of the synergistic ratio in the co-fermentation system. It should also be noted that the replicates in this study were technical rather than biological. Each treatment was performed once. All analytical measurements were conducted in triplicate. These measurements used subsamples from the same fermentation unit. This approach does not account for batch-to-batch variability. Such variability is inherent to the fermentation process. This represents an additional methodological limitation.

Future research should therefore focus on four areas:

(1) conducting in vivo feeding trials to evaluate the growth performance and nutrient utilization of fermented forage mulberry in monogastric animals;
(2) elucidating the molecular mechanisms underlying the synergistic interactions between the mixed strains. These analyses may include transcriptional profiling of key enzyme genes and high-throughput sequencing of microbial community succession;
(3) optimizing the fermentation process for large-scale production;
(4) developing stable compound inoculants to improve fermentation reproducibility.

In addition, studies incorporating both single-strain controls are warranted. These studies could further dissect the specific roles of each strain in the fermentation process.

## 5. Conclusions

This study optimized the solid-state mixed-strain fermentation process of whole-plant forage mulberry using *L. plantarum* CF-LJ-4-2 and *P. membranifaciens* CF-B102-1-M. The optimal conditions were 28 °C, 7 days, 30% inoculum dosage, and a solid-to-water ratio of 1:1.2. Under these conditions, the CF degradation rate reached 44.60%, and the contents of CP, TAA, and EE were significantly improved.

The fermented whole-plant mulberry exhibited remarkably elevated in vitro digestibility, with DMD of 72.30% and CP digestibility of 78.60%.

## Figures and Tables

**Table 1:** Factors and levels of the orthogonal experiment.

Level	Factor A (Fermentation Temperature/°C)	Factor B (Fermentation Time/Days)	Factor C (Inoculum Addition Amount/%)	Factor D (Solid-to-Water Ratio)
1	24	7	30	1:1.0
2	28	14	35	1:1.2
3	32	21	40	1:1.4

**Table 2:** Design of the orthogonal experiment.

Level	Factor A (Fermentation Temperature/°C)	Factor B (Fermentation Time/Days)	Factor C (Inoculum Addition Amount/%)	Factor D (Solid-to-Water Ratio)
1	28	7	25	1:1.0
2	32	7	30	1:1.2
3	36	7	35	1:1.4
4	28	14	35	1:1.2
5	32	14	30	1:1.4
6	36	14	25	1:1.0
7	28	21	30	1:1.4
8	32	21	35	1:1.0
9	36	21	25	1:1.2

**Table 3:** Range analysis of the orthogonal experiment.

Item	Group	Factor A (Fermentation Time/Days)	Factor B (Fermentation Temperature/°C)	Factor C (Solid-to-Water Ratio)	Factor D (Inoculum Addition Amount/%)	CF/%
	T1	7	28	1:1.0	25	18.69
	T2	7	32	1:1.2	30	18.82
	T3	7	36	1:1.4	35	18.09
	T4	14	28	1:1.2	35	21.04
	T5	14	32	1:1.4	25	17.91
	T6	14	36	1:1.0	30	18.53
	T7	21	28	1:1.4	30	23.77
	T8	21	32	1:1.0	35	20.32
	T9	21	36	1:1.2	25	20.74
CF	K1	18.533	21.167	19.180	19.113	-
	K2	19.160	19.017	20.200	20.373	-
	K3	21.610	19.120	19.923	19.817	-
	Range R	3.077	2.150	1.020	1.260	-

**Table 4:** Variance analysis of the orthogonal experiment.

Group	Source of Variation	Sum of Squares	Degrees of Freedom	Mean Square	F Value	*p* Value	Significance
CF	Corrected model	76.62	8	9.58	43.55	<0.001	***
	Fermentation time (A)	47.51	2	23.76	108.00	<0.001	***
	Fermentation temperature (B)	18.64	2	9.32	42.36	<0.001	***
	solid-to-water ratio (C)	1.70	2	0.85	3.86	<0.05	*
	Inoculum addition amount (D)	8.77	2	4.39	19.95	<0.001	***
	Error	3.96	18	0.22	-	-	-

Note: “*” indicates a significant difference, *p* < 0.05; “***” indicates an extremely significant difference, *p* < 0.001.

**Table 5:** Comparison of nutrient components between unfermented and fermented feed mulberry.

Item	Unfermented Feed Mulberry	Fermented Feed Mulberry	*p* Value
CF (%)	26.12 ± 0.05	14.47 ± 0.49	<0.01
CP (%)	20.66 ± 0.40	28.50 ± 0.34	<0.01
EE (%)	9.15 ± 0.99	15.45 ± 0.42	0.01 < *p* < 0.05
CA (%)	7.50 ± 0.27	8.80 ± 0.08	<0.001
Carbohydrates (%)	62.69 ± 0.50	47.25 ± 0.72	<0.001

**Table 6:** Comparison of amino acid contents between unfermented and fermented feed mulberry (unit: %).

Item	Unfermented Feed Mulberry	Fermented Feed Mulberry	*p* Value
Asp	1.42 ± 0.04	1.88 ± 0.02	<0.01
Thr	0.42 ± 0.01	0.64 ± 0.01	<0.01
Ser	0.37 ± 0.04	0.63 ± 0.00	<0.01
Glu	1.06 ± 0.03	1.82 ± 0.01	<0.01
Gly	0.58 ± 0.03	1.07 ± 0.02	<0.01
Ala	0.60 ± 0.01	1.05 ± 0.06	<0.01
Val	0.61 ± 0.01	0.89 ± 0.03	<0.01
Met	0.05 ± 0.06	0.17 ± 0.04	<0.05
Ile	0.39 ± 0.09	0.70 ± 0.05	<0.01
Leu	0.80 ± 0.03	1.23 ± 0.06	<0.01
Tyr	0.22 ± 0.00	0.38 ± 0.04	<0.01
Phe	0.55 ± 0.03	0.79 ± 0.00	<0.01
His	0.23 ± 0.04	0.29 ± 0.00	<0.05
Lys	0.52 ± 0.01	0.63 ± 0.02	<0.05
Arg	0.63 ± 0.04	0.71 ± 0.04	<0.05
Cys	0.02 ± 0.00	0.03 ± 0.02	<0.05
Pro	0.55 ± 0.04	0.83 ± 0.00	<0.01
TEAA	4.07 ± 0.11	6.11 ± 0.09	<0.01
TNEAA	4.66 ± 0.08	7.66 ± 0.07	<0.01
TAA	8.73 ± 0.13	13.77 ± 0.12	<0.01
TEAA/TAA	0.47 ± 0.01	0.44 ± 0.01	<0.05
TEAA/TNEAA	0.87 ± 0.02	0.79 ± 0.01	<0.01

Note: Essential Amino Acids (EAAs): Threonine (Thr), Valine (Val), Isoleucine (Ile), Leucine (Leu), Phenylalanine (Phe), Methionine (Met), Histidine (His), Lysine (Lys), Arginine (Arg). Non-essential Amino Acids (NEAAs):Aspartic acid (Asp), Serine (Ser), Glutamic acid (Glu), Glycine (Gly), Alanine (Ala), Tyrosine (Tyr), Proline (Pro), Cysteine (Cys).

**Table 7:** Results of in vitro simulated rumen digestion.

Digestibility Index	Raw Sample (%)	Fermented Sample (%)	*p* Value
In vitro DMD	61.00 ± 1.90 ^a^	72.30 ± 2.10 ^b^	<0.01
In vitro CP digestibility	64.20 ± 2.30 ^a^	78.60 ± 1.80 ^b^	<0.01

Note: Values are expressed as mean ± standard deviation. a,b Within the same row, different superscript letters indicate significant differences between the raw and fermented samples (*p* < 0.01).

## Data Availability

The original contributions presented in this study are included in the article. Further inquiries can be directed to the corresponding authors.
